# Mobile applications to detect hearing impairment: opportunities and challenges

**DOI:** 10.2471/BLT.18.227728

**Published:** 2019-09-03

**Authors:** De Wet Swanepoel, Karina C De Sousa, Cas Smits, David R Moore

**Affiliations:** a Department of Speech-Language Pathology and Audiology, University of Pretoria, Private Bag X20, Hatfield 0028, South Africa.; b Amsterdam Public Health research institute, Vrije Universiteit Amsterdam, Amsterdam, Netherlands.; c Communication Sciences Research Center, Cincinnati Children’s Hospital, Ohio, United States of America.

Hearing loss affects close to 1.3 billion people and is a growing global health concern as the fourth leading contributor to years lived with disability.^1^ The global economic cost associated with hearing loss is estimated at 750 billion United States dollars (US$) annually.^2^ However, hearing loss has limited public health support, especially in low- and middle-income countries, where incidence is high and resources often scarce and unequally distributed. Consequences of unaddressed hearing loss include impaired communication and reduced psychosocial well-being. Hearing loss also has close links to dementia as one of the primary modifiable risk factors.^3^ The 2017 World Health Assembly Resolution *Prevention of deafness and hearing loss* calls on hearing health-care stakeholders to develop and implement strategies for improved service provision, especially in low- and middle-income countries.^2^

While half of all hearing losses can be prevented, most of the remainder will benefit from significantly reduced functional impairment made possible through early detection and treatment.^2^ Capitalizing on advances in mobile phone penetration, mobile health (mHealth) solutions, that is, use of mobile and wireless technologies for health, provide an opportunity for hearing health service-delivery models that can improve access to and uptake of care.^3^^,^^4^ Greater access to mobile technology is associated with improvements in quality of life, as evidenced by the relationship between mobile connectivity levels and progress towards meeting sustainable development goals.^5^ For low- and middle-income countries, accessible mHealth solutions are particularly appealing for hearing loss prevention, because a very high proportion of people with hearing loss in these countries remains untreated due to poor access to hearing services.^3^ Smartphone applications (apps) are starting to provide alternative accessible hearing tests in two broad categories: clinical (medically regulated) and consumer apps.

## Clinical apps

Clinical hearing assessment apps typically determine hearing sensitivity across tones of different pitch.^6^ Such testing requires adherence to international equipment and calibration standards for medical devices. Health-care providers typically use these assessment apps as alternative audiometers that offer the advantages of affordability and of being mobile, with touchscreen functionality. Validated clinical tools, which are often only available directly from app providers (not on app stores) include iPad- and smartphone-based testing with calibrated headphones.^7^

Examples of community-based screening programmes for children and adults in low- and middle-income countries have used inexpensive, clinically certified smartphone test solutions with calibrated headphones.^7^^,^^8^ Several unique mHealth app features address the lack of formally trained screeners, the complexity of traditional test equipment and the poor surveillance that characterizes screening programmes.^7^^,^^8^ These features include (i) simple, graphic-rich user experiences allowing community members to facilitate screening;^8^ (ii) surveillance of test operators, based on the percentage of correct selection of random no-stimulus presentations; and (iii) monitoring environmental noise compliance.^7^^,^^8^ Since these tests rely on behavioural responses, however, they cannot be used on young infants or other difficult-to-test populations without assistance. Coupling portable objective hearing test technologies that measure physiological responses to sound (for instance, otoacoustic emission or auditory-evoked potentials) with smartphones may make affordable newborn hearing screening possible in low- and middle-income countries.^8^

## Consumer apps

Many consumer apps claim to provide accurate tests on Android and iOS operating systems^6^ employing either tones or speech stimuli to assess hearing ability. Tone-based apps typically implement a variant of the international gold standard hearing assessment, pure tone audiometry. However, since audiometric calibration of headphones and equipment cannot be supported by consumer apps, accuracy is limited.^6^ Furthermore, few tone-based consumer apps have any supporting peer-reviewed evidence, which makes them liable for misuse by uninformed persons.^6^ Those apps that have been evaluated demonstrate wide-ranging variability in accuracy depending on the degree of hearing loss, and phone and headphone type.^6^ Most apps with reasonable accuracy are on the iOS platform, where standardized Apple hardware and software contributes to more uniform results. Apple has recently released a standardized pure-tone audiometry test module with calibration values for their earphones as part of its research software framework (ResearchKit), as well as a subscription hearing aid app (hearingOS). These types of industry developments could ensure more standardized consumer audiometry testing in the future. Unfortunately, due largely to costs, Apple smartphones have poor penetration in low- and middle-income countries and these apps are incompatible with other smartphones. Moreover, the tests are only valid when standard Apple earphones are used.

Speech-based consumer apps typically assess users’ ability to recognize speech in noise. Unlike pure tone audiometry, these tests do not require calibration, allowing use across various devices and headphones.^9^^,^^10^ Speech-in-noise tests directly evaluate what people with hearing loss find most challenging, understanding speech in acoustically challenging environments. These tests are thus considered more representative of impairment in functioning than pure tone audiometry.^10^ In 2016, a national speech-based hearing test (hearZA®, HearX group, Pretoria, South Africa) was launched as a smartphone app (iOS and Android) in South Africa, followed by a version in the United States of America in 2018 (hearScreen USA, American Academy of Audiology, Reston, USA). These digits-in-noise tests present recorded digit triplets (for example, 4–2–7) in background speech-shaped noise to determine the signal-to-noise ratio where a person can identify 50% of triplets correctly. Digits-in-noise tests have previously proven to be effective as national self-screening tests using landline telephone and on internet platforms.^9^ Highly correlated with pure-tone audiometry, digits-in-noise tests’ sensitivity and specificity are up to 90% and can be completed in three minutes.^10^

The World Health Organization recently adopted this approach for the hearWHO app (iOS and Android) released on World Hearing Day 2019. This app has a new version of the digits-in-noise test and uses antiphasic digit stimuli that make the app sensitive to a range of hearing losses including sensorineural, conductive and asymmetrical losses.^11^ In addition to offering a self-test for consumers, this test version could also improve digits-in-noise tests used for school^11^ and other community-based screening programmes. Since the test is language-dependent, it requires development and validation in different languages for widespread global uptake.

## Opportunities and challenges

Advantages of mobile-based digital hearing test solutions (both consumer and clinical), include accessibility, affordability, advanced sensors and software-based quality control, alongside integrated cloud-based data management.^7^^,^^8^ Some apps allow tracking hearing status over time and can also be linked to decision-support resources that encourage users to act on hearing loss. The next question regarding continuum of care is what to do once a person is identified as having hearing loss. One of the principles in screening is that facilities for diagnosis and treatment should be available. While screening apps usually serve only to detect possible hearing loss, with provision of information on causes and treatment options, some include location-based referral options in partnership with audiological societies.^7^ Most people with hearing loss, however, reside in regions that have minimal access to hearing professionals.^3^ Traditional options for hearing aids are therefore either unavailable or unaffordable. Future alternatives using smartphone apps, informed by results from an app-based test, could turn the smartphone into an accessible intervention device for amplification or assistive listening. These apps may reduce perceived barriers in compliance and adoption of hearing aids and can support informational counselling and integrated listening strategies.

A growing concern around health apps is the unstandardized approach to reporting and issues surrounding data security.^4^^,^^12^ Guidelines to report on mHealth interventions, such as the mHealth evidence reporting and assessment checklist,^4^ are becoming increasingly important for comparability and quality of evidence.^4^ In terms of data security, mHealth apps are also targeted for patient data theft, as is the case for electronic health record systems. Vendors and providers do not always ensure that their apps are compliant with security requirements in their jurisdiction. Clinical smartphone apps for medical evaluations require health data security specified in the regulatory process required for medical certification. mHealth apps for hearing tests must be evaluated against their intended use, consumer or clinical, and be scrutinized for data security and privacy before use.^4^^,^^12^

Rapid global advancement in connectivity and technology is changing the landscape of affordable health-care access.^12^ Increasing options are becoming available for consumers and clinicians who use mobile apps to detect, diagnose, and even treat hearing loss. As these technologies become more available, identifying those apps that have been validated for consumer and clinical purposes,^4^ while prioritizing access to follow-up services and data security, will be essential.
